# Impact of Raising Staff Awareness on Recording Patient Consent to Receive Text Message Reminders of Appointments and Increasing the Frequency of Reminders on Did Not Attend (DNA) Rates in Community Mental Health (CMH): A Quality Improvement Project

**DOI:** 10.1192/bjo.2024.388

**Published:** 2024-08-01

**Authors:** Gonjan Kaur, Edelyne Tandanu, Prem Ojha

**Affiliations:** King's College London, London, United Kingdom

## Abstract

**Aims:**

Patients not attending appointments without letting the service know prior (referred to as did not attend – DNA) is a significant problem in community mental health (CMH). However, there are limited studies conducted in the United Kingdom on this issue. Patients forgetting appointments was a reoccurring reason for DNAs in the literature. To address this, we aimed to assess the impact of raising staff awareness on recording patient consent to receive text message reminders of appointments and increasing the frequency of reminders on DNA rates in Arndale House (a CMH service covering Dartford, Gravesend and Swanley as part of the Kent and Medway NHS and Social Care Partnership Trust – KMPT).

**Methods:**

DNA rates at Arndale House from August to October 2023 were assessed to determine a baseline before implementing interventions. Following this, two interventions were put in place; the first occurred on 18/10/23, consisting of an online teaching session for the staff at Arndale on documenting patients’ consent to receive text message reminders for their appointments. Posters with instructions on this were posted on the trust intranet and set up within the building. The second intervention occurred on 20/11/23 and included sending out text message reminders more frequently, from three and one day prior to appointments to seven days beforehand as well. DNA rates for November were analysed to assess the impact of intervention one and December for intervention two. Patient characteristics were also examined monthly to identify any trends among those who DNA. The analysis comprised 109 patients (69 for pre-intervention, 27 for intervention one, and 13 for intervention two).

**Results:**

Pre-intervention DNA rates were 13.4%, 17.5%, and 13.5%, respectively. The incidence of DNA increased to 19.9% after intervention one. However, this was lower than November 2022. The rate for intervention two was 11.6%, lower than that of December 2022. Being White, having a mood disorder and having mental health disorders which fell under more than one category were prominent among those who DNA.

**Conclusion:**

Increasing the frequency of text message reminders of appointments had a significant impact on reducing DNA rates, highlighting a potential intervention which can be implemented in CMH to tackle the issue of DNAs.

